# Poloxamer-Based Mixed Micelles Loaded with Thymol or Eugenol for Topical Applications

**DOI:** 10.1021/acsomega.3c08917

**Published:** 2024-05-20

**Authors:** Jana Sedlarikova, Magda Janalikova, Pavlina Egner, Pavel Pleva

**Affiliations:** † Department of Fat, Surfactant and Cosmetics Technology, Faculty of Technology, Tomas Bata University in Zlin, Vavreckova 275, 760 01 Zlin, Czech Republic; ‡ Department of Environmental Protection Engineering, Faculty of Technology, Tomas Bata University in Zlin, Vavreckova 275, 760 01 Zlin, Czech Republic

## Abstract

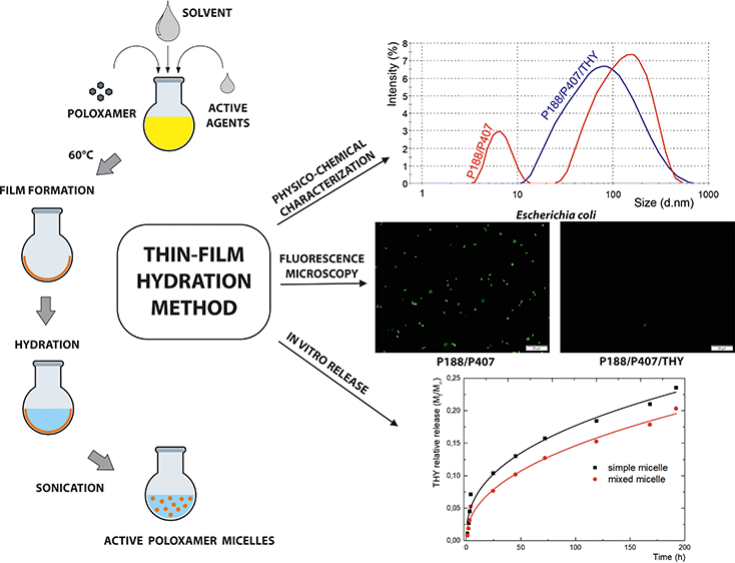

Poloxamers (P184, P188, and P407) have been investigated as the carrier system for eugenol or thymol. A synergic effect of mixed Poloxamers was proved by enhanced micellar parameters, with a lower critical micelle concentration (about 0.06 mM) and the highest surface adsorption of 9 × 10^–7^ mol m^–2^ for P188/P407. Dynamic light scattering revealed a decrease in micellar size after loading with biomolecules. Three mathematical models were applied to study the release kinetics, of which Korsmeyer–Peppas was the best fitted model. Higher relative release was observed for Poloxamer/eugenol samples, up to a value of 0.8. Poloxamer micelles with thymol were highly influential in bacterial reduction. Single P407/eugenol micelles proved to be bacteriostatic for up to 6 h for *S. aureus* or up to 48 h for *E. coli*. Mixed micelles were confirmed to have prolonged bacteriostatic activity for up to 72 h against both bacteria. This trend was also proven by the modified Gompertz model. An optimized P188/P407/eugenol micelle was successfully used as a model system for release study with a particle size of less than 30 nm and high encapsulation efficiency surpassing 90%. The developed mixed micelles were proved to have antibiofilm activity, and thus they provide an innovative approach for controlled release with potential in topical applications.

## Introduction

**Figure 1:**
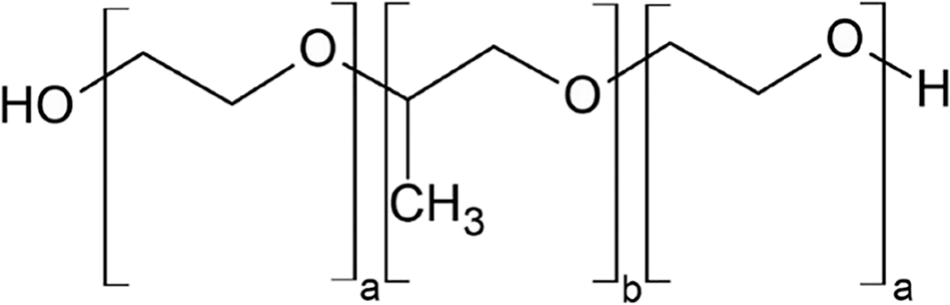
Structure of the Poloxamer.

Phenolic compounds are naturally occurring substances with a higher count of hydroxyl groups linked to aromatic and heterocyclic rings.^1^ Eugenol (4-allyl-2-methoxyphenol) belongs to natural phenolic compounds found mostly in cinnamon and clove essential oils.^2^ Thymol (2-isopropyl-5-methylphenol) is the main component of thyme oil extracted from*Thymus vulgaris* L., belonging to natural terpenoid phenol derivatives.^3,4^ Both of these bioactive molecules are known for their antioxidant, antimicrobial, and anti-inflammatory properties.^5,6^ Their clinical applications are, however, limited by low solubility, absorption, and bioavailability. Some of them exhibit worse physical and chemical stability^7^ and high sensitivity to oxidation and hydrolysis mechanisms, resulting in fast degradation.^8^ Therefore, encapsulation into various carrier systems, such as liposomes and nanoemulsions, has been studied as an attractive strategy to overcome these limitations.^9^ Polymeric micelles represent an attractive thermodynamically stable delivery system that enhances the bioavailability of poorly soluble active agents. These aggregates can be easily fabricated from different types of polymer surfactants, including Poloxamers. These biocompatible polymers^10^ belong among the poly(ethylene oxide)-poly(propylene oxide)-poly(ethylene oxide) (PEO–PPO-PEO) triblock copolymer surface active agents (Figure 1) that can be used for the preparation of suitable carriers with an outer hydrophilic chain and inner hydrophobic core in an aqueous medium.^11,12^ Compared to traditional low molecular weight surface active agents, Poloxamer molecules contain long chains with an M_w_ of thousands g/mol. Poloxamer aggregates can form micelles, reversed micelles, and lyotropic liquid crystals, including lamellar, hexagonal, and cubic aggregates. Due to their structure, they exhibit several exceptional characteristics, such as low critical micelle concentrations, minimal cytotoxicity, and high solubilization capacity, which can be effectively used in the cosmetic, medical, or food industry.^13,14^

The study aimed to prepare and characterize the polymer micelles based on three Poloxamer types or their binary mixtures of different HLB and Mw values as potential carriers for hydrophobic phenolic active compounds. The effect of specific carrier composition on the physicochemical and antibacterial properties of micellar systems and the potential synergism in binary Poloxamer mixed micelles and correlation with the release kinetics study have been investigated.

## Materials and Methods

Poloxamers of various molecular weights and HLB values (Table 1), Tween 80, thymol, and eugenol were supplied by Sigma-Aldrich (Prague, Czech Republic).

**Table 1:** Characteristics of Poloxamer Block Copolymers^a^

	Poloxamer 184	Poloxamer 188	Poloxamer 407
abbreviation	P184	P188	P407
molecular weight	2900	8400	12600
PO/EO ratio	1.11	0.19	0.33
HLB	12–18	29	22

a PO, propylene oxide; EO, ethylene oxide; HLB, hydrophilic lipophilic balance.

Microorganisms (*Escherichia coli* ATCC 25922 and *Staphylococcus aureus* ATCC 25923) were obtained from the Czech Collection of Microorganisms (CCM, Czech Republic). The bacterial cultures were grown on nutrient agar (Himedia Laboratories, India) at 37 °C/24 h. Both bacterial strains are biofilm-positive.^15^

### Preparation of Poloxamer/Phenol Micelles

Poloxamer base micelles were prepared by a thin hydration method by weighing the appropriate amount of Poloxamer or their mixtures in a ratio 1:1 (1% w/v) with eugenol (EUG) or thymol (THY; 0.5% w/v) and dissolving in ethanol. After homogenization (magnetic stirrer, 500 rpm, 30 min), the mixture was evaporated by rotary vacuum evaporation at 50 °C and 55 rpm (Hei VAP Advantage, Heidolph Instruments GmbH & Co. KG, Schwabach) and left in the dark for 24 h at laboratory temperature to remove the residual solvent. The resultant thin film was rehydrated in demineralized water in an ultrasonic bath (40 °C, 40 to 60 min), after which the samples were filtrated via a VWR syringe filter (1.2 μm).

Empty Poloxamer micelles were prepared by weighing the appropriate amount of Poloxamers or their mixtures in a ratio 1:1 (1% w/v), dissolving in demineralized water, and homogenizing under continuous stirring on a magnetic stirrer for 30 min at 500 rpm.

### Characterization of Micellar Parameters of Poloxamer Particles

Micellar properties were analyzed by surface tension measurement using the Wilhelmy plate method (EasyDyne tensiometer K20, Krss GmbH, Germany) at 25 ± 1 °C on diluted Poloxamer samples (0.01–0.5% w/v). The result was an average of five measurements by the instrument.

Gibbs micelle energies were calculated for each formulation according to eq 1(16):
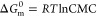
1where CMC is the critical micelle concentration.

The properties of surface active agents in solutions are controlled by the tendency to minimize contact of their hydrophobic chains with water. This phenomenon is achieved by interphase adsorption and aggregate formation. The relationship between surface activity, concentration, and surface adsorption is given by the Gibbs adsorption isotherm, eq 2:
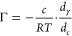
2where Γ is the concentration of the adsorbate at the phase boundary, *c* is the concentration of the surfactant, and γ is the surface tension.

The surface area that is occupied by one surfactant molecule *a*_1_^s^ can be calculated by eq 3:
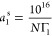
3where *N* is the Avogadro constant.

Interaction parameters were evaluated according to Rubingh’s theory, which is used for systems deviating from the ideal behavior. The following equations 4, 5 enable one to calculate the composition of the mixed micellar aggregate^17^:
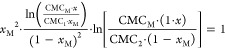
4where *x*_M_ is the molar fraction of the surfactant in a mixed micelle, *x* is the molar fraction of the surfactant in the system, CMC_1_ and CMC_2_ are the values of critical micelle concentrations of individual surfactants, and CMC_M_ is the critical micelle concentration of the mixture.

The interaction parameter β, indicating the deviation of the system from ideality (β = 0), is calculated employing the following eq 5:
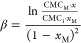
5The negative values of β indicate that interactions between the components of the mixed micellar aggregate are less repulsive; higher negative β values give evidence of attractive forces between surfactant molecules in a mixed micelle.^17^

### Stability of Poloxamer Particles

Physical stability was analyzed by particle size and zeta potential measurement on a Zetasizer Nano ZS device (Malvern Instruments, Ltd., UK). The samples were diluted with demineralized water filtrated via a VWR syringe filter (pore size of 0.45 μm). The size measurement was performed by laser diffractometry at a 90° scattering angle. Zeta potential was measured using folded capillary cells (Malvern Instruments, Ltd., UK) in automatic mode, in adherence with the Smoluchowski model. All measurements were performed on the day of the preparation and after 3 months (storage at 4 °C) at 25 ± 1 °C in triplicate.

### In Vitro Release Study

The release of phenolic compounds from Poloxamer micelles was analyzed by the dialysis method. Poloxamer/phenol solutions were introduced into a Spectra/Por 2 dialyzing membrane, 12 to 14 kDa (Repligen Corporation, Rancho Dominguez, USA) that was inserted into phosphate buffered/Tween 80 solution (pH 7.5) and kept at 25 ± 1 °C under gentle agitation. At defined time intervals, samples were withdrawn, and the released amount of bioactive compound was analyzed by UV–vis spectrophotometry (at 283 nm for thymol and 286 nm for eugenol) using the calibration curves (*y* = 10.184*x* + 0.0112, *R*^2^ = 0.9964 for thymol and *y* = 12.243*x* + 0.0422, *R*^2^ = 0.9888 for eugenol). Following the nonlinear regression analysis using the least-squares method, various kinetic models (the first order, Higuchi, Korsmeyer–Peppas) have been applied to evaluate the release mechanism from Poloxamer micellar particles employing the following equations 6, 7, and 8.

First-order kinetics
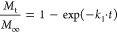
6Higuchi kinetics model
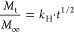
7Korsmeyer–Peppas model of kinetics
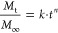
8where *M*_t_/*M*_∞_ represents the fractional drug release at time *t* and *k*_1_, *k*_H_, and *k* represent the first-order release constant, Higuchi constant, and Korsmeyer–Peppas constant, respectively. An exponent *n* characterizes the diffusional release kinetic mechanism. The data were analyzed for the initial 60% release only. The values of *k*_1_, *k*_H_, *k*, and *n* were determined by fitting the release data into respective equations.

### Encapsulation Efficiency and Drug Loading Capacity

Encapsulation efficiency was evaluated by diluting the sample with ethanol/water (1:1) in the test tube that was centrifuged at 5000 rpm for 10 min (Hettich EBA 20, Andreas Hettich GmbH & Co. KG, Germany). A supernatant was then analyzed for the active substance amount by UV–vis spectrophotometry (at 284 nm for thymol and 286 nm for eugenol) using the calibration curves (*y* = 9.7958*x* + 0.0089, *R*^2^ = 0.9901 for thymol and *y* = 14.515*x* + 0.0715, *R*^2^ = 0.9915 for eugenol in ethanol/water). Encapsulation efficiency (%EE) and drug loading (%DL) were then calculated using eqs 9, 10:
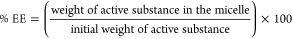
9
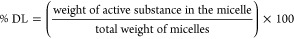
10

### Antibacterial Activity

#### Disk Diffusion Method

The antibacterial activity of selected Poloxamer/phenol samples was tested against Gram-negative and Gram-positive bacteria using the agar disk diffusion method. Sterile paper disks (diameter 6 mm, Whatman, UK) were loaded with 10 μL of the sample, after which they were placed on agar plates previously inoculated with 1 mL of 0.5 McF turbid suspension of bacteria in sterile saline solution. The antibacterial tests were also performed with pure active substances (thymol and eugenol at 0.5% w/v) dissolved in 5 mL of 96% ethanol. The resultant inhibition zones around the samples were recorded, and all tests were performed ten times.

#### Bacterial Growth Kinetics

To examine the change of bacterial growth kinetics with Poloxamer samples loaded with eugenol or thymol, the microplate wells were filled with 200 μL of Mueller Hinton Broth (Himedia Laboratories Pvt. Ltd., Mumbai, India), 10 μL (0.025% v/v) or 5 μL (0.0125% v/v) of Poloxamer sample or without (control bacterial growth), and 5 μL of 0.5 McF turbid bacterial inoculum (*E. coli* or *S. aureus*). The microplate was incubated with shaking at 37 °C for 72 h. The absorbance values (in nine rounds) were read as optical density (OD_600nm_) every half hour with an Infinite 200Pro microplate reader (Tecan, Männedorf, Switzerland).^18^ The modified Gompertz equation was used to describe the bacterial growth kinetics, the lag phase of bacterial growth, to evaluate the antimicrobial effect of the Poloxamer combination. A nonlinear regression analysis (Levenberg–Marquardt algorithm) was used for the calculation of the parameters μ_max_, λ, and *A* for the following conditions: μ > 0, λ > 0, and *A* > 0. The maximum specific growth rate (μ_max_) and asymptotic value are given by eq 11:
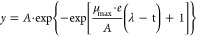
11where μ_max_ is the maximum specific growth rate (h^–1^); λ is the lag phase (h); and *A* is the asymptote defined as the maximum value of relative microorganism counts (−).^19^

#### Cultivation Assay

Bacteria *E. coli* and *S. aureus* were used to determine the antibacterial activity of Poloxamer samples. An overnight culture of each strain was prepared in BHI broth (Oxoid, Basingstoke, UK) at 37 °C. The inoculum was diluted in ratio 1:50 with sterile broth (control) or broth with Poloxamer (Poloxamer/broth ratio was 1:15), without bioactive compounds P407 and P188/P407 as control and with bioactive compounds P407/THY, P407/EUG, P188/P407/THY, and P188/P407/EUG in the final concentration 0.03125% v/v. The first samples were collected immediately (0 h), and tubes were placed at 37 °C. After 1, 3, 6, 24, and 72 h, the samples were withdrawn, and the total viable bacterial counts (CFU mL^–1^) were determined by the automatic Spiral Plater Eddy Jet (IULmicro, New York, USA) on nutrient agar (HiMedia Laboratories Pvt., Ltd., Mumbai, India). The same collected samples were used for the following fluorescence microscopy method.

#### Fluorescence Microscopy

Fluorescence microscopy was performed to confirm the antibacterial activity of Poloxamers against the tested bacteria. To observe an inhibition effect, 2 mL of 0.8% agarose gel (Merck, Germany) was poured on the microscopic glass slide. After the gel solidified, 0.5 μL of the tested solution with microorganisms was applied at 0, 1, 3, 6, 24, and 72 h. A LIVE/DEAD BacLight Bacterial Viability Kit (Thermo Fisher, Waltham, MA, USA), based on the protocol 1, was executed using slight modifications.^20^ SYTO 9 dyed plasma membranes of all bacteria, while propidium iodide could color DNA of only dead cells. The excitation/emission maxima for these dyes are 480/500 nm for the SYTO 9 stain and 490/635 nm for propidium iodide. Thus, bacteria with intact cell membranes stain fluorescent green, whereas bacteria with damaged membranes (dead) stain fluorescent red. Fluorescence microscopy was carried out on an Olympus BX53 fluorescence microscope (Olympus, Tokyo, Japan) equipped with a DP73 Microscope Digital Camera 325 (Olympus, Tokyo, Japan).

#### Antibiofilm Activity

Microtiter plate assay (96-well) was used to determine biofilm production by *Staphylococcus aureus* and *Escherichia coli* with Poloxamer samples. Each well was filled with 195 μL of BHI broth (Brain Heart Infusion; Himedia, Mumbai, India) + 5% w/w sucrose (Himedia, Mumbai, India); then, 10 or 5 μL of Poloxamers (0.0125, 0.0250, and 0.0313% w/v P407/THY, P407/EUG, P188/P407/THY, P188/P407/EUG) and 5 μL of 0.5 McFarland (1 × 10^8^ CFU mL^–1^) turbidity bacterial suspension (*E. coli* and *S. aureus*) were added. The whole plate was incubated at 37 ± 1 °C with no shaking for 72 h in an Infinite 200Pro spectrophotometer (Tecan, Männedorf, Switzerland) for biofilm formation. After cultivation, planktonic cells in microplate wells were rinsed thoroughly with distilled water. Before staining with crystal violet by the Christensen method, the biofilm was fixed by subjecting it to 200 μL of 96% ethanol (Penta, Praha, Czech Republic) for 20 min and pouring it out. The microplate wells were stained with 200 μL of crystal violet for 20 min. After the wells were washed twice with water, the biofilm was solubilized with 200 μL of 96% ethanol (Penta, Praha, Czech Republic).^15^ Each experiment was done in 12 wells to repeat. The blank absorbance values were used to identify whether a biofilm formation was present. The wells higher than the OD value of the blank well were considered to be biofilm producing. Wells containing only BHI with sucrose were used as negative controls.^18^

### Statistical Analysis

Obtained data were presented as mean ± SD using MS Office Excel software (Microsoft, 2020). One-way analysis of variance (ANOVA) using Statistica software (version 10, StatSoft, Inc., Tulsa, OK, USA) at the significance level of *p* < 0.05 was used for the statistical analysis.

## Results and Discussion

### Micellar Parameters of Poloxamer Particles

Micellar characteristics of surfactant systems play a crucial role in their further practical applications. Micellar and interaction parameters analyzed by the tensiometry measurements are summarized in Table 1. Critical micelle concentrations (CMC) of tested individual Poloxamers showed the lowest value of 0.1 mM for P407, then slightly increasing to 0.6 mM for P184, revealing the trend inversely proportional to the Poloxamer molecular weights. It is known that the critical micelle concentration values are significantly affected by applied measurement techniques, specific surfactant type, purity, potential contaminations, etc. Bąk et al.,^21^ who investigated micellar parameters of Pluronic 68 (P188 in our study) by tensiometry, reported a CMC value of about 0.04 mM. An isothermal titration calorimetry method showed the CMC value of Pluronic F127 at 0.34 mM,^22^ which is slightly higher in comparison with data of our study obtained for the P407 sample. A synergic effect of prepared mixed micelles was proved when the critical micelle concentration (CMC) values were significantly lower than those of simple aggregates. The minimum surface tension ranged from 37 to 45 mN·m^–1^, with the lowest value for the P188/P407 sample. It is known that surface active molecules tend to form micellar aggregates spontaneously, which leads to negative Gibbs micellar energies. The results in Table 1 show the values ranging from −22 to −29 kJ mol^–1^ with no confirmed unambiguous favorable effect of combined aggregates on the micellization process. The lower values were observed in Poloxamer with the highest molecular weight (P407); the same trend was confirmed in the study of Pluronic mixed micelles loaded with hydrophobic drugs clozapine and oxcarbazepine.^23^ On the other hand, a positive trend is reflected by the surface concentration at the interphase, Γ values, that increased in the samples based on mixed micelles (up to 9 × 10^–7^ mol m^–2^ in the case of P188/P407).^23^ The surface area occupied by one surfactant molecule has significantly (*p* < 0.05) decreased (from 5 to 2 nm^2^) in mixed micelles, demonstrating tighter organization of Poloxamer molecules at the phase boundary and more effective adsorption. All Poloxamer samples showed negative deviations from ideal behavior, which indicates a mild synergic effect (values β, Table 2). The values of *x*_M_ were only slightly different from the theoretical mixture composition (*x* = 0.5).

**Table 2:** Micellar and Interaction Parameters of the Poloxamer Samples^a^

sample	CMC [mM]	γ_min_ [mN m^–1^]	Δ*G*_m_ [kJ mol^–1^]	Γ × 10^–7^ [mol m^–2^]	*a* [nm^2^]	β	*x*_M_
P184	0.55 ± 0.01^a^	37.4 ± 0.2^a^	–24.10 ± 0.11 ^a^	4.2 ± 0.2^a^	5.0 ± 0.2^a^		
P188	0.17 ± 0.05^b^	45.0 ± 0.4^b^	–22.18 ± 0.13^b^	3.6 ± 0.6^a^	5.1 ± 0.3^a^		
P407	0.10 ± 0.01^b^	38.7 ± 0.4^c^	–28.54 ± 0.18^c^	4.0 ± 0.1^a^	4.2 ± 0.1^b^		
P184/P188	0.04 ± 0.01^c^	40.3 ± 1.8 ^c^	–25.10 ± 0.82^d^	5.0 ± 0.4^b^	3.3 ± 0.2^c^	3.68 ± 0.23^a^	0.40 ± 0.01^a^
P184/P407	0.06 ± 0.01^d^	38.5 ± 2.5^a,c^	–24.10 ± 0.76^a,d,e^	6.8 ± 0.7^c^	2.5 ± 0.3^d^	3.23 ± 0.09^b^	0.39 ± 0.01^a^
P188/P407	0.07 ± 0.01^d,e^	36.7 ± 1.9^a,c^	–23.71 ± 0.19^e^	9.2 ± 0.5^d^	1.8 ± 0.1^e^	1.72 ± 0.22^c^	0.35 ± 0.01^b^

a Different letters indicate significant differences between the samples (*p* < 0.05).

Synergic effects were also shown by Patel et al.,^24^ investigating the mixed micelles based on P123 and F127 combined with different types of significantly hydrophobic copolymers (containing 10% PEO and a variable amount of PPO). Another study proved the synergism between Poloxamer 407 and polysorbate surface active agents (polysorbate 20, 60, 80, 85). It was shown that sorbate surfactants with longer and more unsaturated chains ensured tighter arrangement in the aggregate core, as well as stronger interactions.^17^

### Stability of Poloxamer Particles

**Figure 2:**
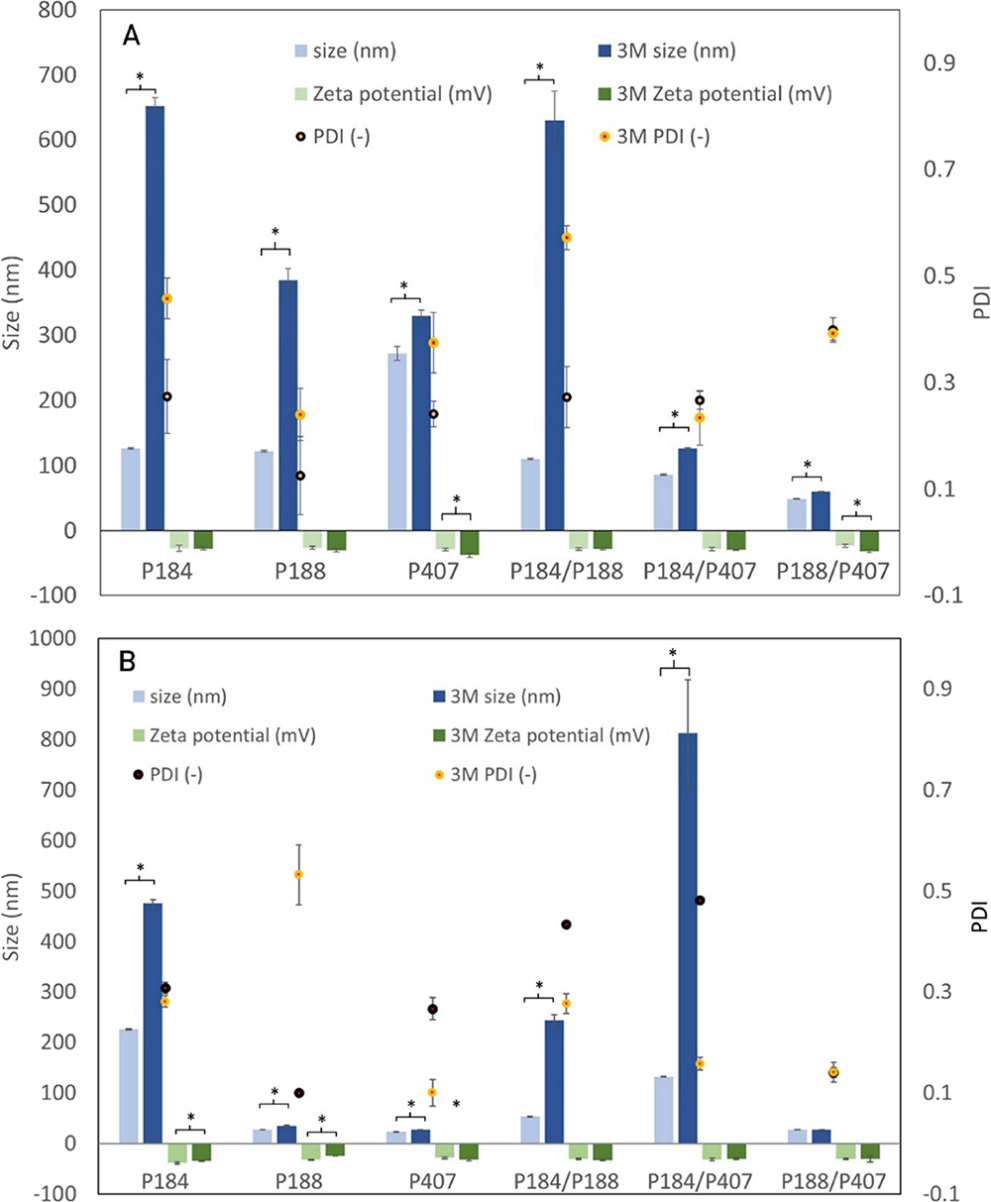
Particle size, polydispersity, and zeta potential of Poloxamer samples with (a) thymol and (b) eugenol. * indicates statistically significant differences, *p* < 0.05.

Particle size is an important characterization parameter affecting the functionality of carrier systems.^3^ The measurements of both empty (Table 3) and loaded (Figure 2) micelles were performed on the day of preparation and after 3 months (storage at 4 °C) to verify the physicochemical stability of the samples. The appearance of the prepared samples is shown in the Supporting Information (Figures S1 and S2). The data in Table 3 show a significant (*p* < 0.05) decrease of particle size in Poloxamer binary mixtures P188/P407 and P184/P407, compared to simple aggregates, with the lowest value of 109.5 nm for P184/P407. The results obtained after 3 months of storage revealed the significant (*p* < 0.05) change of particle size in almost all the samples, except P188, with the most prominent increase (almost 60%) for the P184/P407 binary mixture. An opposite trend, i.e., decreased micelle size, was observed in the P184 and P188 samples (see Table 3).

**Table 3:** Particle Size and Polydispersity of Unloaded Poloxamer Samples^a^

sample	the day of preparation		after 3 months	
	particle size [d nm]	PDl	particle size [d.nm]	PDl
P184	383.3 ± 14.2^a^	0.5 ± 0.0^a^	253.6 ± 41.5 ^a,b*^	0.6 ± 0.1 ^a^
P188	297.5 ± 70.9^a^	0.7 ± 0.2^a,b^	244.0 ± 16.6 ^a^	0.7 ± 0.1 ^a,b^
P407	149.4 ± 10.2^b^	0.4 ± 0.1^a^	237.4 ± 60.9 ^a,b*^	0.8 ± 0.2 ^a,b,c^
P184/P188	178.0 ± 39.0^b^	0.6 ± 0.0^b^	414.7 ± 51.7 ^c*^	0.8 ± 0.1 ^a,b^
P188/P407	112.4 ± 10.9^c^	0.4 ± 0.0^c^	201.6 ± 17.3 ^b*^	1.0 ± 0.1 ^c^
P184/P407	109.5 ± 13.3^c^	0.6 ± 0.2^a,b,c^	271.3 ± 13.2 ^a*^	0.6 ± 0.2 ^a^

a Different letters in the same column and * in the same line indicate significant differences between the samples (*p* < 0.05).

Incorporating bioactive compounds (eugenol and thymol) into Poloxamer micelles caused changes in measured size according to the specific combination of Poloxamer–bioactive molecules. A significant decrease in values was monitored in micelles loaded with phenols in most cases, compared to their empty counterparts (Figure 2). Encapsulation of eugenol or thymol probably caused an enhancement of hydrophobic interactions in the micellar cores, resulting in improved compactness. A similar trend was reported in the study of Vivero-Lopez et al.,^25^ investigating Pluronic F127 micelles loaded with resveratrol. On the other hand, an increase in the hydrodynamic micelle diameter was observed after the encapsulation of hydrophobic drugs clozapine and oxcarbazepine due to the aggregate swelling.^23^ Moreover, the authors presumed solubilization in the inner (PPO) and outer (PEO) layers of mixed micelles.

Within the stability testing after 3 months of storage, the significant increase (*p* < 0.05) of particle size indicating the aggregation process was observed in all Poloxamer/THY samples, of which the binary mixtures P184/P407 and P188/P407 revealed the highest stability (Figure 2a). The latter-mentioned system proved relatively high resistance to aggregation also in the case of eugenol, even with regard to the PDI index of 0.13, which has not almost changed after the observed storage time (Figure 2b). The polydispersity index of other tested samples has not exceeded 0.5. On the other hand, the most prominent size increment (84%) was noticed in the P184/P407/EUG mixture (Figure 2b). Distribution curves were also analyzed in order to determine the potential effect of a mixed micellar system on the particle size and polydispersity. Regarding the favorable data for the P188/P407 micelle, this formulation was selected and compared with a single P407 micelle (see the Supporting Information, Figures S3 and S4). A different behavior was revealed in the thymol and eugenol samples. In the case of thymol, a wider distribution of particles was observed in mixed micelle (P188/P407) compared to the single component aggregate (P407). On the other hand, a mixed micelle exhibited better stability in time (Figure S3). Eugenol-loaded particles showed a narrow size distribution, both for single and mixed micellar aggregates. In general, all eugenol samples showed good stability in time (Figure S4).

The entrapment efficiency and drug loading of eugenol or thymol into Poloxamer micellar aggregates are summarized in Table S2. The tested samples exhibited high encapsulation efficiency exceeding 90% and drug loading based on the weight/weight calculation ranged from 11.6 to 12.6%. There were no statistically significant differences among the individual Poloxamer samples.

Zeta potential measurements were carried out to analyze the surface charge and stability of the prepared Poloxamer micellar aggregates. The negative potential up to −31 mV (not shown here) was obtained for unloaded particles, although Poloxamers belong among nonionic compounds. Negative values of zeta potential were also observed by Tănase et al.^26^ who investigated the effect of hydrophobicity of selected Poloxamers on the physical and antimicrobial properties of curcumin-loaded micellar systems. Zeta potential is known to be affected by the factors, such as pH, ionic strength, and the presence of various additives in the media. Adsorption of these compounds can shift the position of the shear plane from the particle surface. Presumably, a negative charge could also occur as the consequence of some ionic contaminants, some of which can also be surface active. Encapsulation of eugenol or thymol did not significantly affect the surface charge (Figure 2). Negative values, which can be due to repulsive interactions among the micellar aggregates, will ensure high physical stability without a tendency to aggregate.^27^ It was also reported in the literature that negative charges can prolong the release kinetics of the carrier system. On the other hand, particles with neutral zeta potential are not affected by the pH of the specific media, and this target site can be easily reached. Based on obtained zeta potential data, EUG-loaded micelles showed more stable particles with the most significant value of −39 mV for the P184 sample. Zeta potential values were not significantly changed in binary Poloxamer mixtures after storage time.

### In Vitro Release of Active Substances from Poloxamer Micelles

**Figure 3:**
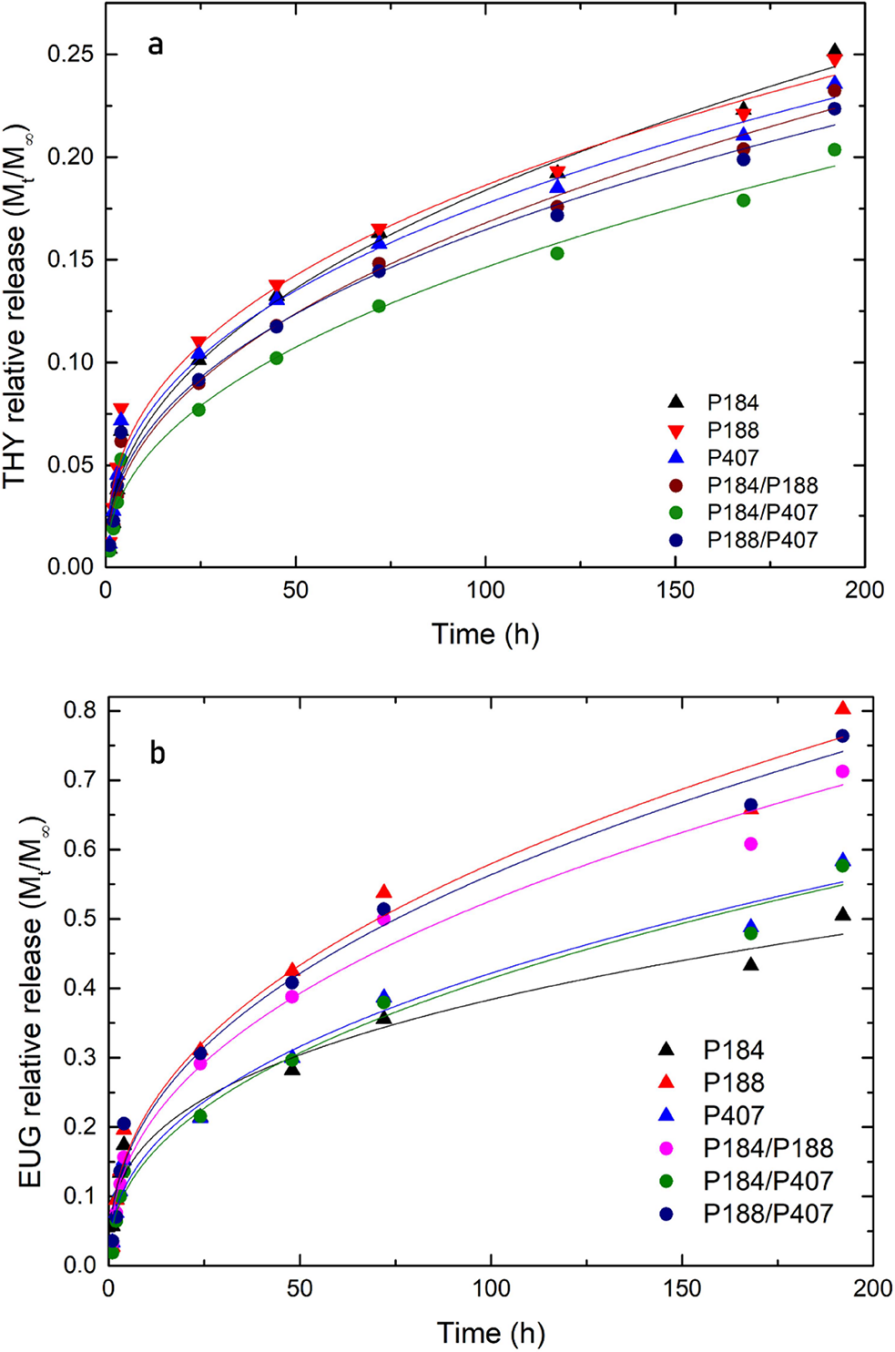
In vitro relative release of (a) thymol (THY) and (b) eugenol (EUG) from Poloxamer micelles. The Korsmeyer–Peppas (KP) model fits of the data are shown as solid lines.

An investigation on the release kinetics conditions from the carrier systems has significant importance, reflecting the further in vivo applications.^5^ The data of THY and EUG relative release from single and mixed Poloxamer micelles (Figure 3) show a similar trend, including a faster initial release (up to 24 h) of bioactive molecules, followed by a slowdown within the further observed time interval. However, the maximum released amount differed significantly when less than 0.3 relative release was revealed in the case of thymol samples, whereas almost 0.8 relative release was monitored in micelles loaded with eugenol. The correlation between the release kinetics and hydrophobicity of encapsulated actives has been reported in the literature.^28^ Although both THY and EUG represent hydrophobic compounds, they differ in molecular structure and aqueous solubility, leading to different release trends. External factors, such as the temperature and relative humidity, must also be considered. In the study of Zhu et al., who investigated poly(lactide-*co*-glycolide)-based microparticles loaded with thymol, an enhancement in release amount at increased temperature and humidity was observed.^29^

A slower release kinetics was observed in mixed micelles loaded with thymol, whereas the same trend was not confirmed in the eugenol samples. It is known that the release process can proceed via various mechanisms, such as diffusion or swelling or deformation of polymer carriers. A similar trend, i.e. faster initial release, followed by decelerating, was observed for Pluronic micelles loaded with Nimodipine drug by Sotoudegan et al.^30^ The highest release was monitored from F68, corresponding to P188 in our study. Contrary to our samples, however, significantly faster kinetics were observed in the study of Nimodipine (almost 100% was released after 10 h from the F68 micelle). It was also reported that the release rate indirectly correlates with polymer concentration, HLB value, and molecular weight. The highest released amount was shown in the Poloxamer systems with higher hydrophilicity.

Three kinetic models (the first order, Higuchi, Korsmeyer–Peppas) were considered to describe the release mechanism of thymol and eugenol from Poloxamer-based micelles (Tables 4 and 5). The Korsmeyer–Peppas model is usually applied for predicting the release mechanisms from polymer-based systems and the Higuchi model is often used to describe especially the release from Poloxamer-based carriers.^31,32^ Based on the coefficient of determination values, the Korsmeyer–Peppas (KP) kinetic model best fitted the release profiles of phenolic molecules from Poloxamer micelles. Based on the *n* constant in Tables 4 and 5 (mostly 0.39–0.45), a release process according to Fickian diffusion from the spherical or cylindrical shapes of Poloxamer aggregates can be predicted. A range up to *M*_t_/*M*_∞_ < 0.6 is recommended for the release data evaluation, which was valid in our measurement, except for selected eugenol data at the highest observed time.^33,34^

**Table 4:** Rate Constants and Coefficients of Determination Using Kinetic Models for the Release of Thymol (THY) from Poloxamer Samples

	first order		Higuchi		KP		
sample	*K* (h^–1^)	*R*^2^	*K*_H_ (h^–1^)	*R*^2^	*K*_H_ (h^–1^)	*R*^2^	*n*
P184	0.0243	0.8827	0.0182	0.9764	0.0249	0.9829	0.4343
P188	0.0196	0.9253	0.0184	0.9494	0.0313	0.9770	0.3875
P407	0.0238	0.8919	0.0198	0.9654	0.0291	0.9795	0.3923
P184/P188	0.0183	0.9209	0.0167	0.9784	0.0222	0.9833	0.4396
P184/P407	0.0203	0.8980	0.0145	0.9805	0.0189	0.9841	0.4454
P188/P407	0.0175	0.9210	0.0163	0.9663	0.0245	0.9796	0.4138

**Table 5:** Rate Constants and Coefficients of Determination Using Kinetic Models for the Release of Eugenol (EUG) from Poloxamer Samples

	first order		Higuchi		KP		
sample	*K* (h^–1^)	*R*^2^	*K*_H_ (h^–1^)	*R*^2^	*K*_H_ (h^–1^)	*R*^2^	*n*
P184	0.0264	0.7629	0.0573	0.9641	0.0838	0.9757	0.4199
P188	0.0206	0.9122	0.0378	0.8815	0.0816	0.9691	0.3362
P407	0.0191	0.8940	0.0417	0.9649	0.0620	0.9780	0.41655
P184/P188	0.0214	0.9374	0.0521	0.9715	0.0752	0.9831	0.4226
P184/P407	0.0190	0.9133	0.0558	0.9681	0.0815	0.9802	0.4200
P188/P407	0.0202	0.9173	0.0410	0.9720	0.0565	0.9791	0.4325

### Antibacterial Activity

#### Disk Diffusion Method

**Figure 4:**
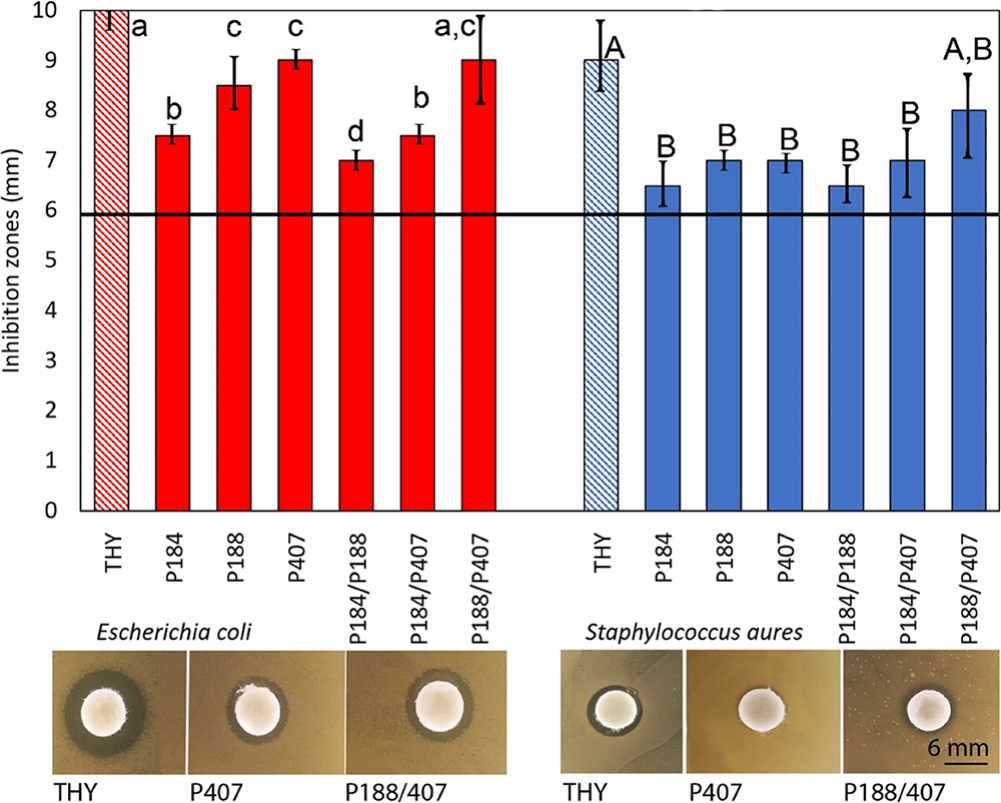
Inhibition zones in millimeters of Poloxamer samples with thymol. The horizontal line indicates a diameter of disk (6 mm). Different lowercase and uppercase letters indicate significant differences between the pure thymol and Poloxamer samples against *E. coli* and *S. aureus*, respectively (*p* < 0.05).

The disk diffusion method was used to evaluate the antibacterial properties of prepared Poloxamer samples against *S. aureus* and *E. coli* bacteria. As monoterpenoid phenols with an active hydroxyl group, eugenol and thymol are known for antibacterial, antifungal, anti-inflammatory, and antioxidant properties. Eugenol inhibits some bacterial enzymes and contributes to cell membrane disruption,^35^ similar to thymol.^36^ None of the tested Poloxamer samples with eugenol proved any inhibition by this method, whereas the antibacterial activity of Poloxamers loaded with thymol was observed. Thymol mode of action against *S. aureus* is probably targeting bacterial aldo-keto reductase.^37^ The results (Figure 4) show the inhibition zones measured as diameter, including a 6 mm disk. It was proved that free thymol is the most active against *S. aureus* and *E. coli* compared to this bioactive compound encapsulated into micellar aggregates. Among Poloxamer samples, the biggest inhibition zones were noticed by mixing P188/P407/THY against both tested bacteria. Obviously, thymol loaded into our Poloxamer samples was able to disturb the outer microbial membrane, leading to a permeability increase.^38^ In the case of the other single or mixed Poloxamers, a significantly lower (*p* < 0.05) activity was shown. Thus, thymol encapsulated in Poloxamer micelles showed smaller inhibition zones than pure thymol, corresponding to the theoretical presumption of its slower release into media due to encapsulation.

#### Bacterial Growth Kinetics

**Figure 5:**
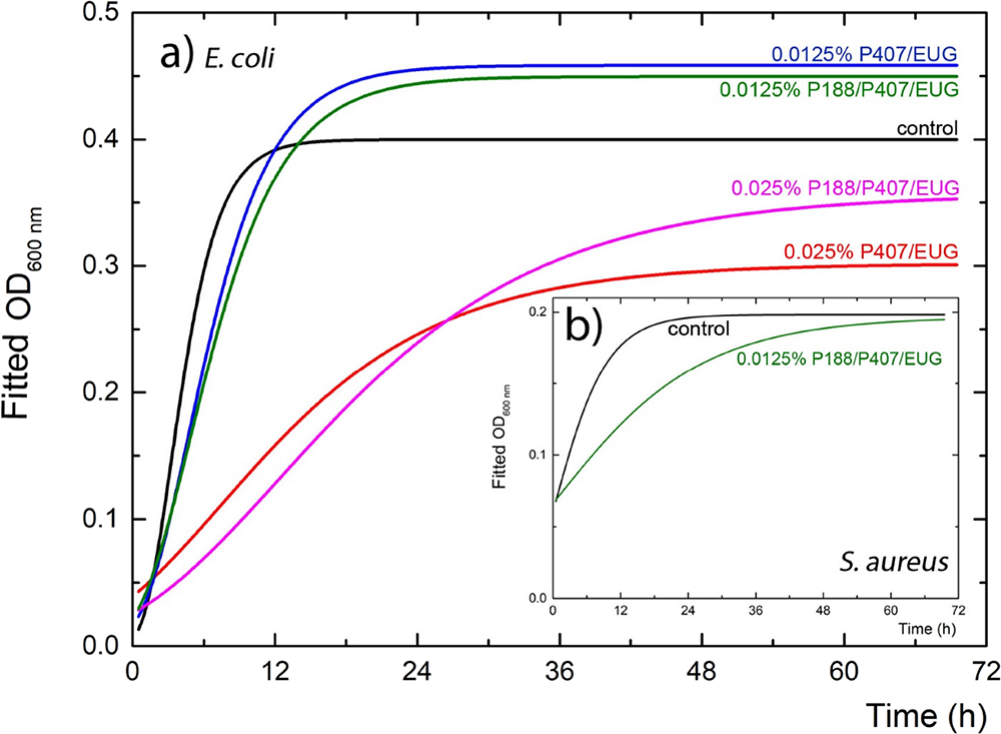
Growth kinetics of bacterial species: (a) *E. coli*; (b) *S. aureus* in the absence and presence of Poloxamer/EUG samples. Lines represent a fitted model according to the Gompertz equation. Indicated concentrations represent the concentration of eugenol. Missing curves were from experiments with no *S. aureus* growth.

The comparison of *E. coli* and *S. aureus* growth curves with two concentrations of Poloxamers only with eugenol is plotted as optical density (OD_600_) over time analyzed by the Gompertz method, Levenberg–Marquardt algorithm (Figure 5). The population growth kinetics is described by growth parameters (λ, μ_max_). Low λ values indicate that bacterial strain can rapidly grow (short lag phase), while increased λ values indicate the antibacterial effect. The maximum growth rate (μ_max_) is defined as the maximum rate of cell population (OD_600_) increase per unit time (h^–1^).^19^

In this experiment, all Poloxamer samples with thymol reduced bacterial growth (*E. coli*, *S. aureus*) at a concentration of 0.0125% v/v. In the literature, the minimal inhibitory concentration (MIC) value for free thymol against *Escherichia coli* was measured at 188 μg mL^–1^ (equivalent to 0.0188% v/v) against *Escherichia coli* (chicken isolate),^39^ which is higher but comparable to our achieved results. Similarly, Zhou et al. described the MIC value for pure thymol against *S. aureus* ATCC25923 (the same as in this study) to be 200 μg mL^–1^ (equivalent to 0.0200% v/v)^37^ and with a different strain (*S. aureus* ATCC6538) the value was determined even to 140 mg·L^–1^ (equivalent to 0.0140% v/v).^40^ Minor discrepancies might be caused by the intrinsic properties of each tested strain or by the performance of the MIC method.

Bacteria *E. coli* with Poloxamer/EUG samples (Figure 5a) were growing, so the growth curves could be analyzed. It was found that the maximum growth rate (μ_max_) decreased at higher tested concentrations (0.025% v/v) of single P407/EUG and mixed P188/P407/EUG Poloxamer samples in comparison with control growth (Tab. S1). *Staphylococcus aureus* was inhibited by all samples except 0.0125% P188/P407/EUG (Figure 5b), which proves the slowest release of eugenol from mixed Poloxamer micelles compared with single counterparts.

#### Cultivation Assay and Fluorescence Microscopy

Antibacterial activity was tested in two ways—cultivation and microscopy methods. Antimicrobial activity of thymol and eugenol in various Poloxamer formulations was observed at defined time intervals: 0, 1, 3, 6, 24, 48, and 72 h. *Escherichia coli* and *Staphylococcus aureus* only in broth and with pure Poloxamers served as controls.

**Figure 6:**
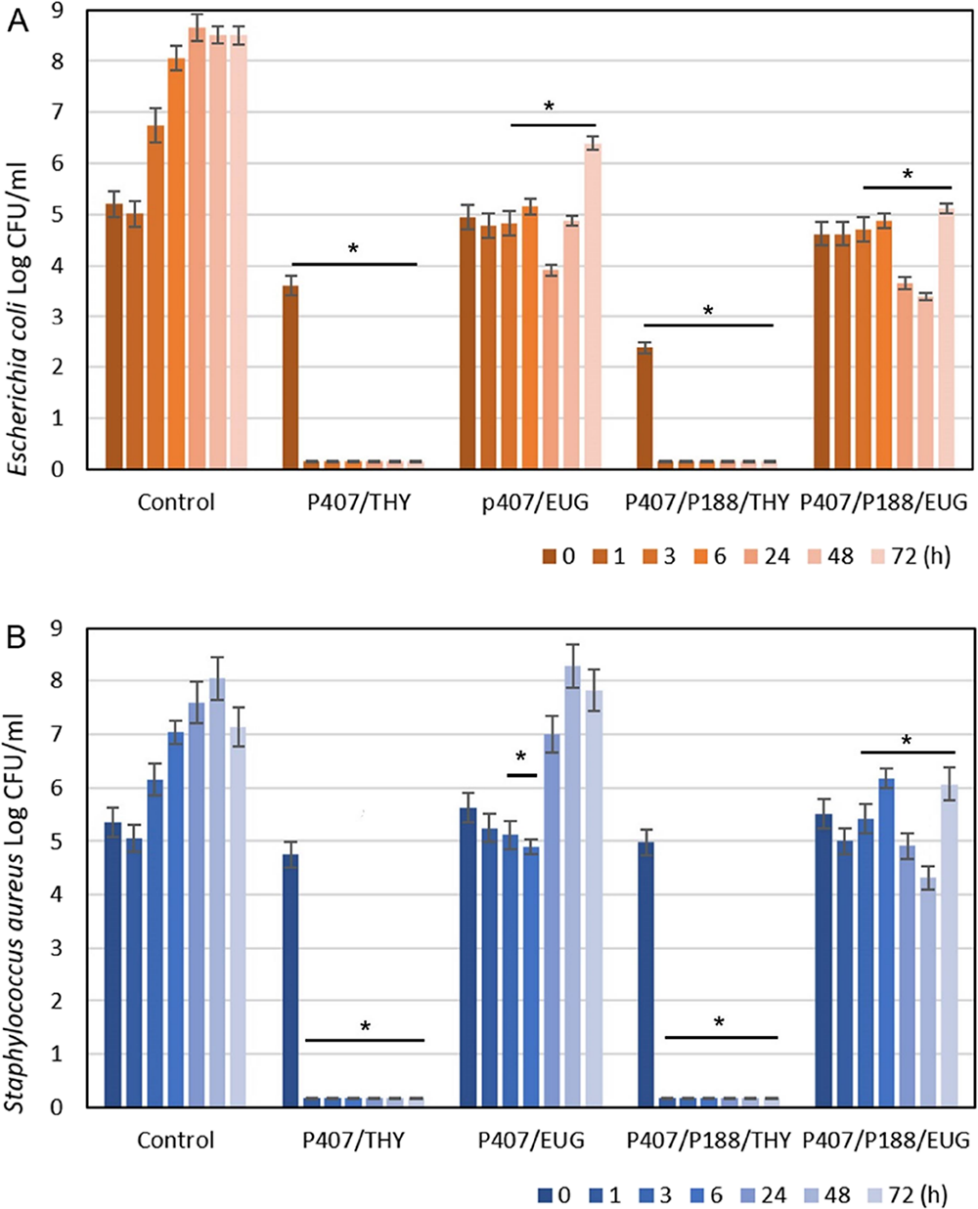
Determination of bacterial counts (Log CFU mL^–1^) during cultivation without (control) or with Poloxamer samples with thymol (THY) and eugenol (EUG) until 72 h for (A) *Escherichia coli* and (B) *Staphylococcus aureus*. Each column with bar represents the mean and standard deviation. Columns marked with the symbol * are statistically different from the growth control at each time interval (*p* < 0.05).

**Figure 7:**
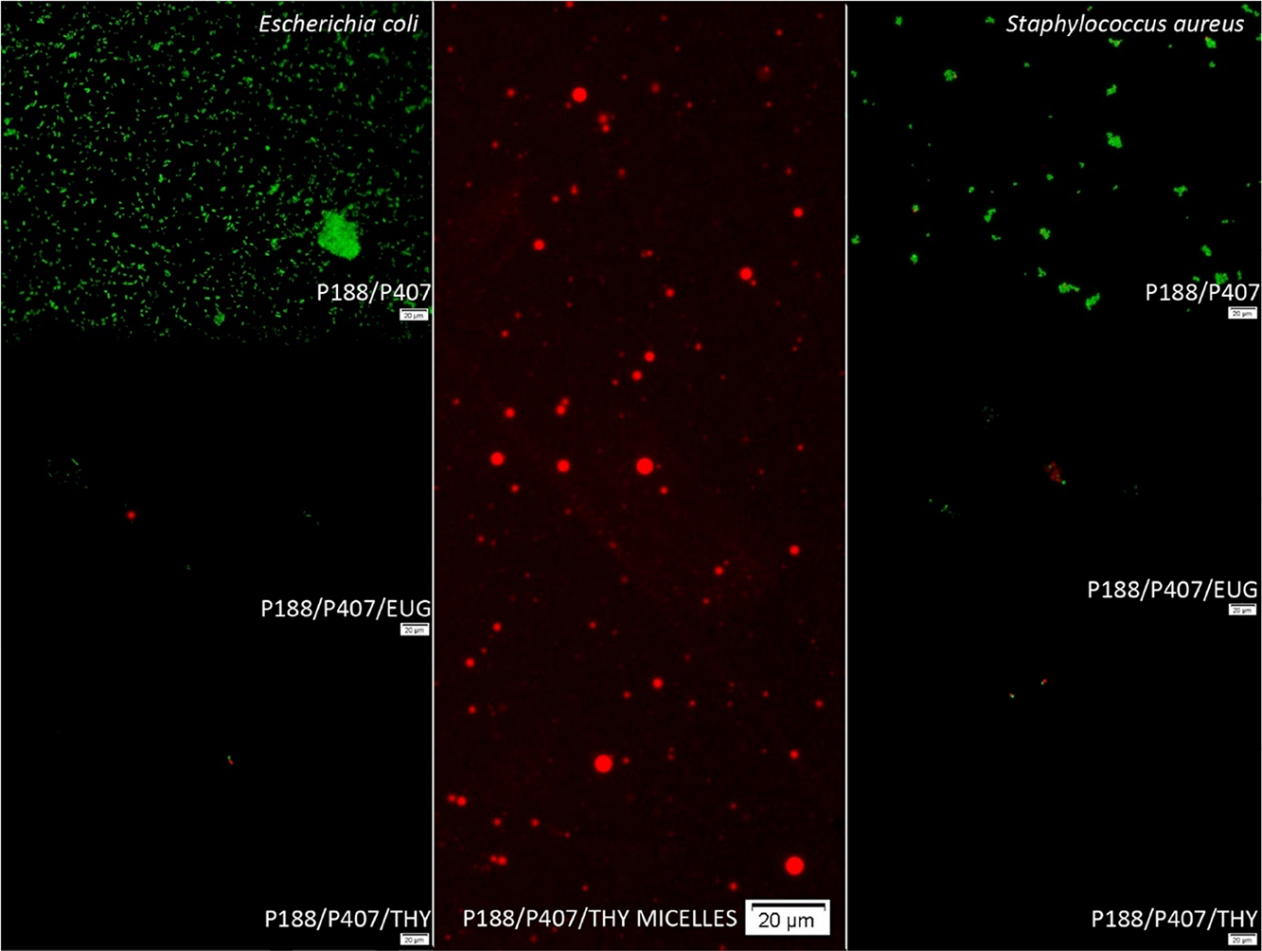
Fluorescence microscopy of mixed Poloxamer micelles before (middle) and after 6 h of cultivation with *E. coli* and *S. aureus*.

Countless living cells were observed by fluorescence microscopy on the agarose surface with P407 and P188/P407 Poloxamers mixed with bacteria, showing no inhibitory activity of pure Poloxamers. Detectable total counts of living cells corresponded to the bacterial counts found by the cultivation method (Figures 6 and 7). Thus, since the results of both methods were comparable, the described fluorescence determination can be used for rapid detection of antimicrobial activity in situ.

At tested concentrations, thymol proved to have a much higher effectivity against Gram-positive bacteria than eugenol, which can also be suggested from their MICs. The MIC value for *S. aureus* was 0.05% v/v and 0.1% v/v for free thymol and eugenol, respectively.^41^ Thymol can be considered an effective agent in both Poloxamer samples (P407; P188/P407). Bacterial counts were expressed as Log CFU mL^–1^ of *Escherichia coli* (A) and *Staphylococcus aureus* (B) during 72 h of incubation without (control) or with P407/THY, P407/EUG, P188/P407/THY, and P188/P407/EUG (Figure 6). It was significantly (*p* < 0.05) proved that both bacterial strains were immediately reduced to a detectable minimum in the presence of either single or mixed Poloxamers with thymol. After 1 h, both bacterial inocula (*E. coli*, *S. aureus*) were completely reduced (5 Log CFU mL^–1^). It can be concluded that thymol was immediately released in sufficient concentration to be described as bactericidal.

Eugenol, a primary component of the clove essential oil, exhibits a broad spectrum of antifungal and antibacterial activity.^42^ The MIC values for free eugenol were determined at 0.05% v/v for *E. coli* and 0.1% for *S. aureus*.^41^ The single Poloxamer samples with eugenol (0.5% w/v) had a bacteriostatic effect on both tested bacteria during the first 6 h of cultivation. After 3 h, *E. coli* was significantly (*p* < 0.05) reduced by more than 1 Log CFU mL^–1^; bacteria started to grow slowly until 72 h, when the counts were similar to the control after only 3 h of cultivation (Figure 6A). On the other hand, the growth of *S. aureus* was significantly reduced (*p* < 0.05) only up to 6 h, following the comparable growth to control (Figure 6B). This study concluded that eugenol was more quickly released from single micelles than from mixed aggregates to get a sufficient concentration for inhibiting *S. aureus* and *E. coli* (Figure 6).

The mixed Poloxamer micelles with eugenol exhibited bacteriostatic activity against *E. coli* and *S. aureus* during 72 h with little reduction in bacterial counts after 24–48 h (Figure 6). Thus, mixed micelles are the best solution for combining the slower and faster release of bioactive compounds. It can be seen from Figure 3 that the same compound, eugenol, was released differently from the P188 and P407 Poloxamers. In P188/P407/EUG mixed micelles, the same release pattern as for a single P188 can be seen. The cultivation experiment proved that eugenol was initially released from P188 micelles, followed by the release from P407 micelles (Figure 6). Other authors^43^ also revealed a higher antimicrobial efficiency of Poloxamer P188 samples compared to P407 due to the faster release of a bioactive compound (propolis). Eugenol can serve as a model system to study the effective release of bioactive compounds encapsulated in Poloxamer-based carriers.

Mixed Poloxamer P188/P407/THY micelles before cultivation with bacteria dyed with propidium iodide can be seen in Figure 7 in the middle. After 6 h of bacterial cultivation with Poloxamers without bioactive compounds, a considerable amount of green bacteria (*E. coli*, *S. aureus*) dyed by SYTO 9 were observed. Cultivation of mixed Poloxamers loaded with bioactive compounds after 6 h revealed a bacteriostatic effect, and only a few dead or alive cells could be detected on a slide with agarose, which means more than 2–4 Log reduction (Figures 6 and 7). Microscopy results correlated with the results from the cultivation method, so the slide agarose method can be used to roughly quantify the bacterial counts and determine the antimicrobial activity of turbid liquid samples.

#### Antibiofilm Activity

The fight against bacterial biofilm formation is an urgent problem in healthcare, food, biomaterials, and biotechnology. The study of Namivandi-Zangeneh et al.^44^ reports the synergistic bactericidal activity against Gram-negative bacteria by synthetic antimicrobial polymers in combination with essential oils, where the antimicrobial polymers play a secondary role as delivery vehicles for essential oils. In this study, the antibiofilm activity of all single and mixed Poloxamer solutions with bioactive compounds was determined by the Christensen method. According to the results of this study, the antibacterial activity of encapsulated thymol and eugenol was proved. Poloxamer samples with thymol at the lowest tested concentration (0.0125% v/v) were bactericidal; therefore, biofilm production could not even be observed. On the other hand, samples with eugenol (0.0125% v/v) allowed both bacteria to grow, but they could reduce biofilm formation by the tested biofilm-positive bacterial strains *S. aureus* and *E. coli*. The study by Garcia-Salinas et al.^45^ declared the antibiofilm activity of free thymol against *S. aureus*.

## Conclusions

Synergic effects of mixed Poloxamer micelles were proved by the lower critical micelle concentrations, Gibbs micelle energy values, and smaller surface area per surfactant molecule compared to single component carriers. Plant-based phenolic compounds, represented by eugenol and thymol, were successfully encapsulated into Poloxamer-based micellar carriers, with the size affected by the formulation composition and the most appropriate stability for the P188/P407 binary mixture. The release profiles differed depending on the specific type of active substance with the lower relative release of thymol from Poloxamer samples.

The antibacterial activity of thymol-loaded Poloxamers was proved by disk diffusion and a bacterial growth kinetics method. A kinetic study supported by the Gompertz model revealed a slower release of eugenol from mixed samples than that from single micelles. Poloxamer/thymol samples showed prominent bactericidal activity even at the lowest tested concentration. In eugenol samples, a longer bacteriostatic activity was shown in mixed Poloxamer micelles, supporting the slower release of active compounds. All Poloxamer/eugenol samples were confirmed to have antibiofilm activity against *S. aureus* and *E. coli*. The conducted in vitro study suggests that the mixture of Poloxamer micelles can serve as a suitable carrier system for the sustainable topical delivery of hydrophobic bioactive compounds with significant potential in addressing biofilm-related issues.

## Supporting Information Available

Stability study: Poloxamer/THY mixed micelles after preparation, after 3 months of storage at 4 °C, Poloxamer/EUG mixed micelles after preparation, after 3 months of storage at 4 °C, distribution curves of Poloxamer samples with thymol, and distribution curves of Poloxamer samples with eugenol; bacterial growth kinetics: rate constants using kinetic models for bacterial species from the Gompertz equation; and encapsulation efficiency and drug loading of Poloxamer/phenol micelles (PDF)
