# Supplementary material for: Poloxamer-Based Mixed Micelles Loaded with Thymol or Eugenol for Topical Applications
Source: ACS Omega. 2024 May 20;9(22):23209–19. doi: 10.1021/acsomega.3c08917 (PMC11154913; doi:10.1021/acsomega.3c08917)
Supplement: Supplementary file 1 — ao3c08917_si_001.pdf [file ao3c08917_si_001.pdf]

Supporting Information

Poloxamer-based Mixed Micelles Loaded with Thymol or Eugenol for Topical Applications

Jana Sedlarikova [1], Magda Janalikova [2], Pavlina Egner [1], and Pavel Pleva [2,*]

1 Department of Fat, Surfactant and Cosmetics Technology, Faculty of Technology, Tomas Bata University in Zlin, Vavreckova 275, 760 01 Zlin, Czech Republic

2 Department of Environmental Protection Engineering, Faculty of Technology, Tomas Bata University in Zlin, Vavreckova 275, 760 01 Zlin, Czech Republic

* Correspondence: ppleva@utb.cz

Figure S1: Poloxamer/THY mixed micelles: a) after preparation, b) after 3 months storage at 4 °C.
Figure S2: Poloxamer/EUG mixed micelles: a) after preparation, b) after 3 months storage at 4 °C.
Figure S3: Distribution curves of Poloxamer samples with thymol.
Figure S4: Distribution curves of Poloxamer samples with eugenol.
Table S1: Rate constants using kinetic models for bacterial species from the Gompertz equation.
Table S2: Encapsulation efficiency and drug loading of Poloxamer/phenol micelles.

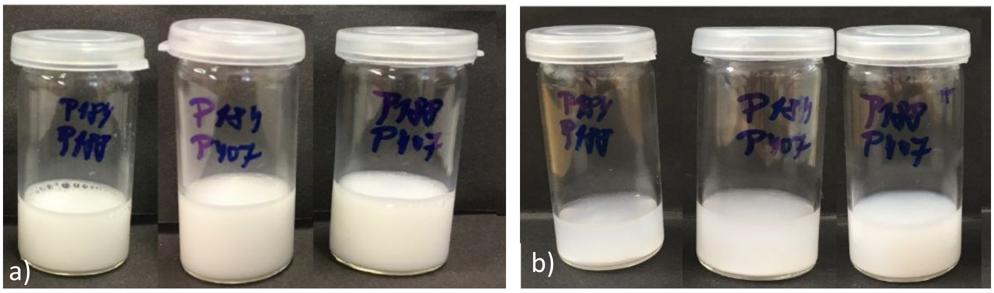

Figure S1. Appearance of Poloxamer/THY mixed micelles: a) after preparation, b) after 3 months storage at 4 °C.

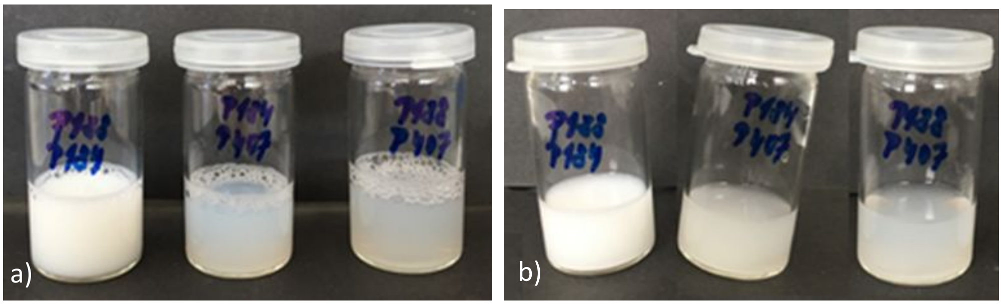

Figure S2. Appearance of Poloxamer/EUG mixed micelles: a) after preparation, b) after 3 months storage at 4 °C.

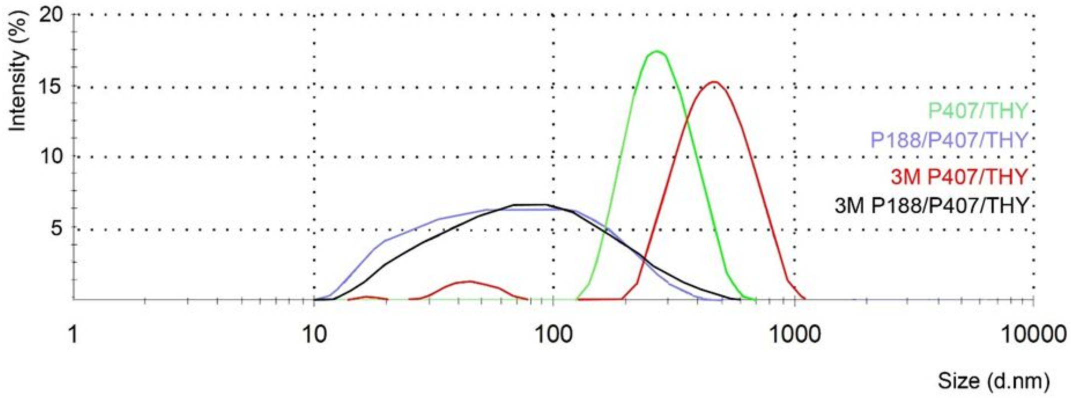

Figure S3: Distribution curves of Poloxamer samples with thymol.

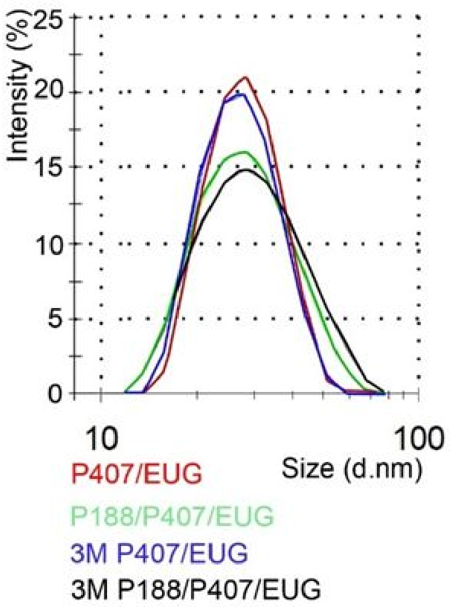

Figure S4: Distribution curves of Poloxamer samples with eugenol.

Table S1: Rate constants using kinetic models for bacterial species from the Gompertz equation.

	Sample	μ_{max} (h^{-1})	λ(h)	R^2
Escherichia coli	**Control**	0.44±0.02	0.39±0.00	0.96
	**0.025% P407/EUG**	0.09±0.01	0.30±0.00	0.95
	**0.0125% P407/EUG**	0.26±0.01	0.47±0.00	0.96
	**0.025% P188/P407/EUG**	0.08±0.00	0.36±0.01	0.95
	**0.0125% P188/P407/EUG**	0.22±0.01	0.45±0.00	0.96
Staphylococcus aureus	**Control**	0.19±0.01	0.20±0.00	0.97
	**0.025% P407/EUG**	*no growth*		
	**0.0125% P407/EUG**			
	**0.025% P188/P407/EUG**			
	**0.0125% P188/P407/EUG**	0.07±0.00	0.20±0.00	0.95

Table S2: Encapsulation efficiency and drug loading of Poloxamer/phenol micelles.

	EE (%)	Drug loading (%)
**P407/THY**	90.48±0.01	12.68±1.57
**P188/P407/THY**	90.90±0.38	12.74±2.40
**P407/EUG**	91.64±2.50	12.51±0.26
**P188/P407/EUG**	92.59±2.99	11.55±0.41
